# Characterizing endophytic competence and plant growth promotion of bacterial endophytes inhabiting the seed endosphere of Rice

**DOI:** 10.1186/s12866-017-1117-0

**Published:** 2017-10-26

**Authors:** Denver I. Walitang, Kiyoon Kim, Munusamy Madhaiyan, Young Kee Kim, Yeongyeong Kang, Tongmin Sa

**Affiliations:** 1 Department of Environmental and Biological Chemistry, College of Agriculture, Life and Environmental Sciences, Chungbuk National University, Cheongju, Chungbuk 28644 Republic of Korea; 2 Temasek Life Sciences Laboratory, Biomaterials and Biocatalyst, National University of Singapore, Singapore, Singapore

**Keywords:** Endophytic competence, Plant growth promotion, Physiological characterization, Rice seeds, Bacterial endophytes

## Abstract

**Background:**

Rice (*Oryza sativa* L. ssp. *indica*) seeds as plant microbiome present both an opportunity and a challenge to colonizing bacterial community living in close association with plants. Nevertheless, the roles and activities of bacterial endophytes remain largely unexplored and insights into plant-microbe interaction are compounded by its complexity. In this study, putative functions or physiological properties associated with bacterial endophytic nature were assessed. Also, endophytic roles in plant growth and germination that may allow them to be selectively chosen by plants were also studied.

**Results:**

The cultivable seed endophytes were dominated by *Proteobacteria* particularly class *Gammaproteobacteria*. Highly identical type strains were isolated from the seed endosphere regardless of the rice host’s physiological tolerance to salinity. Among the type strains, *Flavobacterium* sp., *Microbacterium* sp. and *Xanthomonas* sp. were isolated from the salt-sensitive and salt-tolerant cultivars. PCA-Biplot ordination also showed that specific type strains isolated from different rice cultivars have distinguishing similar characteristics. *Flavobacterium* sp. strains are phosphate solubilizers and indole-3-acetic acid producers with high tolerance to salinity and osmotic stress. *Pseudomonas* strains are characterized as high siderophore producers while *Microbacterium* sp. and *Xanthomonas* sp. strains have very high pectinase and cellulase activity. Among the physiological traits of the seed endophytes, bacterial pectinase and cellulase activity are positively correlated as well as salt and osmotic tolerance. Overall characterization shows that majority of the isolates could survive in 4–8% salt concentration as well as in 0.6 M and 1.2 M sucrose solution. The activities of catalase, pectinase and cellulase were also observed in almost all of the isolates indicating the importance of these characteristics for survival and colonization into the seed endosphere. Seed bacterial endophytes also showed promising plant growth promoting activities including hormone modulation, nitrogen fixation, siderophore production and phosphate solubilization.

**Conclusion:**

Though many of the isolates possess similar PGP and endophytic physiological traits, this study shows some prominent and distinguishing traits among bacterial groups indicating key determinants for their success as endophytes in the rice seed endosphere. Rice seeds are also inhabited by bacterial endophytes that promote growth during early seedling development.

**Electronic supplementary material:**

The online version of this article (10.1186/s12866-017-1117-0) contains supplementary material, which is available to authorized users.

## Background

Plant-microbe interactions have important advantages especially concerning beneficial bacteria. Plants are internally inhabited by plant-associated bacteria, known as endophytes, which directly influence the host plant cells mediating responses as a result of interactions [1] without damaging the host or eliciting strong defense responses [2]. As the plant endosphere niche presents a unique habitat, bacterial endophytes are likely to have differential functions, specialization, adaptations and competence [3, 4]. Furthermore, it is likely that only specialized endophytic strains are capable of colonizing and surviving in the reproductive plant organs [3, 5] and that only bacteria with competitive and adaptive colonization characteristics could inhabit the seeds [4]. Metagenomic analysis of sequences from bacterial endophytes of rice roots predicted properties and metabolic processes important for endophytic lifestyle. These include prominent features such as flagella or motility mechanism, plant-polymer degrading enzymes, protein secretory systems, iron chelation, acquisition and storage, quorum sensing, ROS detoxification, and other characteristics probably involved in the entire nitrogen cycle [6]. Furthermore, comparative genomics of traits relevant to plant colonization and establishment of symbionts, phytopathogens, rhizosphere bacteria and soil bacteria show that there are putative properties that are significantly observed in endophytes compared to other types of bacteria interacting with plants [7] confirming those features studied by Sessitsch et al. [6].

Despite differences in ecological niches, plant growth-promoting rhizobacteria (PGPR) that are free-living and endophytic bacteria utilize some of the same mechanisms to directly and indirectly promote plant growth [8–10]. Plant hosts, on the other hand, may also confer a selective advantage over endophytes that show beneficial associations. As bacteria develop beneficial interactions with plants, they are able to enhance growth and yield, suppress pathogens, and mobilize some micronutrients [11]. Hardoim et al. [7] added that bacterial endophytes also possess other plant growth promoting abilities such as nitrogen fixation, phosphate solubilization and iron chelation. The seeds are also shown to be sources of endophytic bacteria of the rice plant [12] and that rice seed bacterial endophytes rapidly colonize the roots then the shoots of developing rice plants [13] indicating their endosphere competence in terms of colonization, survival and even transmission to the next generation hosts. Mano and Morisaki [14] also observed that the bacterial flora of the seeds is closer to that of the shoots than to those of the roots supporting the results of Hardoim et al. [13]. The communities of rice bacterial endophytes were also seen to actively respond to the changes in the environmental conditions of their host [13] and that bacterial adaptation as well as plant host factors affect the overall community structure of endophytes residing in different rice cultivars [15].

There have been numerous reports on indigenous endophytic bacteria in various plants and plant tissues. However, studies on bacterial endophytes inhabiting rice seeds together with their putative endophytic functional traits and plant growth promotion are few most especially on specific cultivars that are anthropogenically selected based on their potential commercial or special applications. Thus, the purpose of this study was to investigate the endophytic bacterial floras in the different salt-sensitive and tolerant rice cultivars. Also, some prominent features of putatively known functions associated with bacterial endophytic nature that confer an advantage for colonization and survival in the seeds were investigated. In addition, the roles of seed endophytes in germination and plant growth promotion (PGP) that may allow them to be selectively chosen by plants were also studied.

## Methods

### Seed samples

All seeds used in this study were taken from Rural Development Administration (RDA), South Korea. Seed germination started in May and seedling transplantation was done in June. There were five salt-tolerant cultivars included namely IR669646-3R-178-1-1 (FL478), CSR 28 (IC27), IR55179-3B-11–3 (IC31), IR58443-6B-10-3 (IC32), IRRI154 (IC37), and a salt-sensitive cultivar (IR29). The salt-tolerant cultivars: IC27, IC31, IC32 and IC37 are experimental hybrid lines being studied for their salinity tolerance. IR29 is a sodium accumulating line that is salt-sensitive during early and mature plant growth development. FL478 is a hybrid from the salt-sensitive line IR29 and a salt-tolerant sodium excluding Pokkali B [16]. IC27 and IC31 have one common parent, IR4630–22–2-5-1-3 which is also distantly related to Pokkali. One of the parents of IC32 could also be traced to Pokkali. The highly salt-tolerant cultivar, IC37, has parental lines different from the other cultivars. All cultivars in this study belong to *Oryza sativa* ssp. *indica*. All the seed samples were harvested from August to early September (Table 1). All seeds used for bacterial isolation were fresh. Seeds were also stored at 4 °C without any seed treatment for further use.

**Table 1:** Characteristics of the six (6) rice cultivars (*Oryza sativa* L. ssp. *indica*) used to assess bacterial community associated with the seeds

Rice Cultivar	Parental Lines	Harvest Date	Stem Height	Salinity Tolerance	
			cm	Early growth	Mature plant
IR29	IR833–6–2-1-1 I 11 (1561–149-1)//1R1737	21 August 2014	78	Weak	Weak
FL478	IR29/POKKALI B	18 August 2014	61	Strong	Moderate
IC27	IR42/IR 4630–22–2-5-1-3	05 September 2014	82	Moderate	Moderate
IC31	IR 4630–22–2-5-1-3/NONA BOKRA	15 August 2014	71	Strong	Moderate
IC32	AT 401/IR31868–64–2-3-3-3	18 August 2014	69	Strong	Moderate
IC37	IR 73012–137–2-2-2/PSB RC 10 (IR 50404–57–2-2-3	22 August 2014	67	Strong	Strong

Source: International Rice Research Institute, Philippines (IRRI); Rural Development Administration, Korea (RDA)

### Surface sterilization of seeds

Bacterial communities of the rice seed endosphere were assessed by culture-dependent approaches. Surface sterilization of rice seeds was done according to Hardoim et al. [13]. Under sterile conditions, decontaminated forceps were used to remove the hulls of rice seeds (1 g). Subsequent surface-sterilization was done at 30 °C for 25 min in an orbital shaker (200 rpm) with a 50 ml solution containing 0.12% sodium hypochlorite (NaClO) and salts (0.1% sodium carbonate, 3% sodium chloride, and 0.15% sodium hydroxide) [17]. Removal of the surface adhered NaClO was achieved by washing with 50 ml 2% sodium thiosulfate [18] repeated twice at 30 °C for 10 min under orbital shaking (200 rpm). The seeds were rinsed 5–8 times with sterile distilled water before the seeds were subjected to rehydration for at least 1 h at room temperature in 100 ml autoclaved demineralized water. The efficiency of sterilization was confirmed by plating 100 μL of the final rinse onto R2A agar plates and incubating them for 7 days at 28 °C.

### Culturable bacterial population

Surface sterilized seeds were ground with an autoclaved mortar and pestle. Culturable populations of seed endophytic bacteria were determined by counting the colony forming units (CFU) on R2A (DB – Difco) plates using spread plate technique after serial dilution of the homogenized surface sterilized seed samples (1.0 g). Ten-fold serial dilutions were made and 100 μl aliquots were spread onto an R2A agar in three replicates for each dilution. Plates were incubated at 28 °C. For bacteria population, counting was done every 24 h for 6 days. Unique bacteria from each plate were chosen based on colony color and morphology. Identification of the bacterial isolates was done through 16S rRNA gene sequence analysis.

### 16S rRNA gene sequence analyses

Pure cultures of endophytic bacteria were subjected to 16S rDNA sequence analysis. Isolates were grown on nutrient agar plates. Genomic DNA was extracted and PCR was used to amplify the 16S rRNA genes using the primers 27F: 5′-AGA GTT TGA TCC TGG CTC AG-3′ as the forward primer and 1492R: 5′-GTT TAC CTT GTT ACG ACT T-3′ as the reverse primer [19] followed by identification of the 16S rRNA nucleotide sequences using PCR-direct sequencing, via the fluorescent dye terminator method (ABI Prism™ Bigdye™ Terminator cycle sequencing ready reaction kit v.3.1). The products were purified using Millipore-Montage dye removal kit and ran in an ABI3730XL capillary DNA sequencer with a 50 cm capillary. The obtained 16S rDNA sequences were aligned and the affiliations deduced using BLAST analysis in the EzTaxon server (https://www.ezbiocloud.net/) [20]. Phylogenetic analyses were performed using MEGA version 6 [21] after multiple alignments of the data by CLUSTAL W [22]. DNA substitutions were done according to the Jukes and Cantor model [23] and clustering was performed using the neighbor-joining method [24]. The statistical confidence of the nodes was estimated by bootstrapping using 1000 replications [25]. The nucleotide sequences of 16S rRNA genes were deposited to the GenBank® database under accession numbers KY393309-KY393357.

### Screening of PGP characteristics

Production of indole-3-acetic acid (IAA) by the isolates was quantified in the presence and absence of tryptophan [26]. Filter sterilized tryptophan was supplied in the medium at a concentration of 500 μg ml^−1^. The ability to solubilize phosphate (tricalcium phosphate) was carried out in NBRIP-BPB plates [27]. Siderophore production was studied on CAS agar plates prepared according to Alexander and Zuberer [28]. ACC deaminase (1-aminocyclopropane-1-carboxylate deaminase; ACCD) activity was determined by growing the bacterial isolates in nitrogen-free medium amended with 3 mM ACC as nitrogen source [29] and the amount of *α*-ketobutyrate produced by the enzymatic hydrolysis of ACC was estimated following Honma and Shimomura [30]. Nested PCR was performed to amplify *nif*H gene in bacterial endophytes. For the first PCR, PolF/PolR primer set was used, whereas for the nested PCR, *nif*HFor and *nif*HRev primer set was used according to Soares et al. [31].

### Rice seed germination with and without salt stress and early seedling development

Rice seeds, *Oryza sativa* L. ssp. *indica* ‘IR29’ were surface sterilized as described above. Aliquots (100 μl) of water from the final wash were spread on R2A to ensure efficiency of sterilization. Seed treatments consisted of soaking surface sterilized seeds in sterile media (0.03 M MgSO_4_) or late log phase cultures of the isolates for 4 h. At the end of seed treatments, 30 seeds were transferred to each petri plate containing sterile filter papers moistened with 10 ml distilled water and treatments were maintained in triplicates. The plates were then moved to a plant growth chamber maintained at 25 ± 2 °C under 12 h/12 h dark/light conditions. Seed germination was checked every 24 h for 5 days. Additionally, germination tests under 150 mM salt solution with sterile distilled water acting as a control were also conducted. In another set of experiments, treated and non-treated rice seeds were germinated in the same conditions as above. After 2 days, 5 fully germinated seeds were transferred into a moist sterile plant pouch and allowed to continue growing up to 7 days. Root and shoot length as well as wet and dry weights were measured at the end of the experiment.

### Characterization and putative endophytic adaptations of seed bacterial isolates

The strains were initially checked for Gram reaction and colony characteristics, and characterization for oxidase and catalase activities were done following standard methods. The intrinsic resistance of the bacterial isolates against salinity was evaluated by observing growth on NA medium (Nutrient broth – Merck) amended with a final concentration of NaCl (2, 4, 6, 8% *w*/*v*). The plates were incubated for 3–5 days at 28 ± 2 °C. The same experiment was carried out with NaCl amended nutrient broth and OD was recorded after 3 days of incubation. Osmotic tolerance of the bacterial isolates was also evaluated in 5 ml NB broth amended with 0.6 M [12] and 1.2 M [4] sucrose and bacterial growth was measured at 540 nm after 3 days of incubation. Motility of bacteria was observed in semi-solid media prepared according to Tittsler and Sandholzer [32] and modified with 0.005% 2,3,5-triphenyltetrazolium chloride. Cellulase test was studied on carboxymethycellulose (CMC) plates prepared according to Kasana et al. [33] while pectinase activity was studied according to Jacob et al. [34]. Cellulase and pectinase activity were both visualized using Gram’s iodine.

### Genetic diversity by BOX-PCR

Genomic DNA of endophytic bacteria isolated from the seed endosphere of different rice cultivars was amplified directly using colony PCR or by isolating genomic DNA using genomic isolation kit (Promega, USA). The primer used for the BOX-PRC reaction was BOX-AIR (5′-CTACGGCAAGGCGACGCTGACG-3′) as described by Naik et al. [35]. Each reaction contained 2 μL of 10 mM dNTP, 2 μL of 10X PCR buffer, 2 μL of 10 pmol BOX-AIR primer, 0.2 μL Taq polymerase and MilliQ water adjusted to a total volume of 20 μL. Amplification was performed in a PTC200 DNA Thermal Cycler (MJ Scientific, USA) using the following program: 95 °C for 5 min for colony PCR and 3 min for genomic DNA, 30× (94 °C for 3 s, 92 °C for 30 s, 50 °C for 1 min, 65 °C for 8 min), and a final extension of 65 °C for 8 min. Hierarchical cluster analysis was performed using IBM SPSS version 20.

### Statistical analysis

Randomized block design was used for seed germination and early growth development. Data from the results were normalized, subjected to analysis of variance (ANOVA) and mean significant difference were compared using least significant difference (LSD) at *P* ≤ 0.05 using SAS version 9.1 package (SAS Institute Inc., Cary, NC, USA). Data from PGP traits and physiological activities were used to generate the biplot ordination diagram of principal component analysis with Primer V.6.

## Results

### Cultivable Rice seed endophytic community

**Fig. 1:**
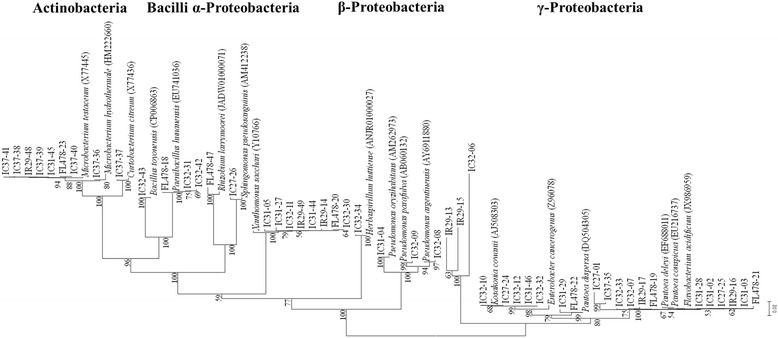
Phylogenetic diversity among bacterial endophytes in the seeds of rice. Neighbor-joining tree based on 16S rDNA gene sequences. Bootstrap values are shown at the branch points

Six rice cultivars grown in the experimental fields of RDA (South Korea) were selected and harvested in August 2014. The population of the culturable bacterial endophytic community of rice seeds was assessed in five salt-tolerant cultivars and one salt-sensitive cultivar after surface sterilization and rehydration up to 10 h. The population density ranges from 4.23 to 6.52 log CFU g^−1^ fresh weight (Table 2). A total of 49 isolates with 18 distinct bacterial strains were identified from the internal tissues of rice seeds. The 16S rRNA gene identification of these cultures revealed that endophytes encompass members of 12 genera within the classes *Alphaproteobacteria, Betaproteobacteria, Gammaproteobacteria*, *Actinobacteria* and *Firmicutes* (Fig. 1). Several genera dominate the seed endosphere including *Flavobacterium*, *Pantoea*, *Microbacterium*, *Xanthomonas*, *Kosakonia*, *Pseudomonas* and *Paenibacillus*. There are common as well as cultivar-specific bacterial isolates from the different rice cultivars. Among the isolates, representatives of *Flavobacterium* sp. were isolated in all the rice cultivars, indicating that it is a common and possible dominant member of the bacterial endophytic community in rice seeds. *Xanthomonas* sp. and *Microbacterium* sp. were also isolated in four rice cultivars (IR29, FL478, IC31 and IC32, and IR29, FL478, IC31 and IC37, respectively) while *Kosakonia* sp. (IC27, IC31 and IC32) and *Paenibacillus* sp. (FL478 and IC32) were isolated in other rice cultivars. Highly identical type strains were isolated from the seed endosphere regardless of the rice host’s physiological tolerance to salinity. The salt-sensitive rice cultivar, IR29, shares some common isolates with other salt-tolerant rice cultivars. Aside from *Flavobacterium* sp., *Microbacterium* sp. was also found in FL478, IC31 and IC37 while *Xanthomonas* sp. was isolated from FL478, IC31 and IC32. These similar type strains found in both the salt-sensitive and salt-tolerant cultivars might indicate other pertinent distinguishing factors that select cultivable bacterial communities in the rice cultivars other than the plant’s physiological adaptation to salt stress.

**Table 2:** Bacterial population profiles in the seeds of salt-tolerant and salt-sensitive cultivars of *Oryza sativa* ssp*. indica*

Rice cultivar	Population	Gram (−)	Gram (+)	Cultivable isolates
	CFU g^−1^	%	%	
IR29	5.46 ± 0.09^c^	42.9	57.1	7
FL478	5.31 ± 0.02^c^	57.1	42.9	7
IC27	6.31 ± 0.04^b^	100.0	0.0	4
IC31	5.64 ± 0.04^c^	90.0	10.0	10
IC32	6.52 ± 0.04^a^	71.4	28.5	14
IC37	4.24 ± 0.04^c^	0.00	100.0	7

Population is presented as means ± SE (standard error) from three replicates. Means with the same letter are not statistically significant

**Fig. 2:**
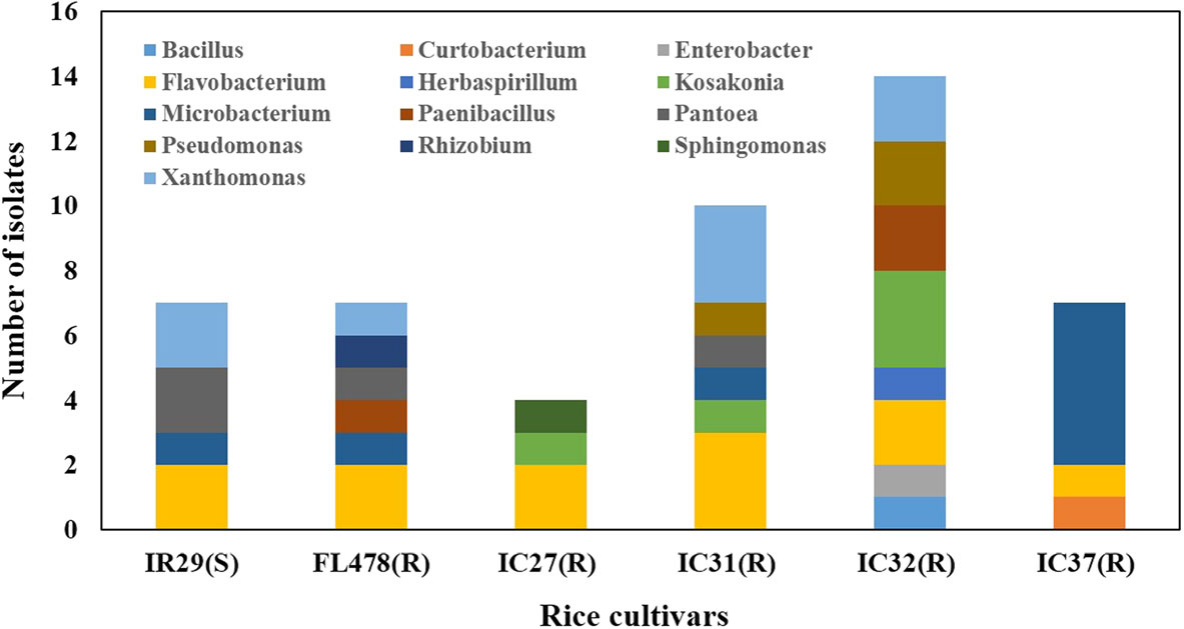
The distribution of the different bacterial genera of endophytes isolated in the different rice cultivars. (S) – salt-sensitive cultivar; (R) – salt-tolerant cultivars

Aside from the shared members of bacterial communities, there were also cultivar-specific isolates such as *Rhizobium larrymoorei*, *Herbaspirillum huttiense* and *Curtobacterium citreum* (Fig. 2). Potentially novel and unidentified bacterial isolates were also present, representing some members of *Enterobacter* and *Pantoea*. Most of the seed endophytes showed high 16S rRNA gene similarities isolated and/or sequenced from the rice phyllosphere, rhizosphere and endosphere, suggesting that these bacteria might be well adapted to the rice niche.

### Genetic diversity of seed bacterial endophytes

To have an idea of the genetic relationship between the isolated endophytes from the different rice cultivars, BOX-PCR fingerprinting method was done (Additional file 1: Figure S2a). DNA fingerprinting via BOX-PCR generated diverse banding patterns with band size ranging from 100 bp to over 3000 bp. For the 49 endophytic bacterial isolates, there were 10 distinct clusters that formed. *Microbacterium* strains formed a major cluster with three subclusters. *Xanthomonas* strains also formed a major cluster with two subclusters. The *Paenibacillus*, *Kosakonia* and *Pantoea* strains also formed their own distinct clusters while *Flavobacterium* major cluster was further divided into four subclusters (Additional file 1: Figure S2b).

### Plant growth promoting characteristics

Different rice cultivars contain bacterial endophytes with multiple PGP characteristics (Additional file 2: Table S1). All of the isolates were able to produce IAA in varying degrees from 2.4–66.0 μg ml^−1^ in the presence of L-tryptophan. ACC deaminase activity, an important PGP trait especially under stress conditions, was observed in 5 isolates (470–7036 nmol α-KB h^−1^ mg protein^−1^) that were cultured from different rice cultivars except for IR29 and IC27. ACCD bacteria include *Pseudomonas* sp. IC31–04, *Paenibacillus* sp. FL478–18, *Kosakonia* sp. IC32–32, *Herbaspirillum* sp. IC32–34 and *Microbacterium* sp. IC37–36; all of which belong to major groups commonly observed as plant growth promoting bacteria. Many of the bacterial isolates could fix nitrogen (33%), solubilize phosphate (73%) and produce siderophores (65%) with varying degrees of metabolic activity in relation to these parameters (Table 3).

**Table 3:** Comparison of plant growth promoting traits and physiological activities observed in different studies of bacterial endophytes

PGP trait or Putative Endophytic Trait	This study	Elbeltagy et al. (2001) [39]	Okunishi et al. (2005) [4]	Kaga et al. (2009) [12]	Palaniappan et al. (2010) [54]	Johnston-Monje and Raizada (2011) [58]	Hardoim 2011 [70]	Xu et al. (2014) [59]	Chimwamurombe et al. (2016) [35]	Average Occurrence
Total isolates tested	49	11	26	78	39	91	20	84	29	
Catalase	49 (100%)	ND	26 (100%)	ND	ND	ND	13 (65%)	ND	ND	88/95 (93%)
Cellulase	45 (92%)	10 (91%)	ND	ND	ND	29 (32%)	8 (40%)	ND	ND	92/171 (54%)
Pectinase	47 (96%)	11 (100%)	ND	ND	ND	28 (32%)	ND	ND	ND	86/151 (60%)
Motility	20 (41%)	11 (100%)	26 (100%)	58 (74%)	ND	ND	12 (60%)	ND	ND	127/180 (71%)
Oxidase	35 (71%)	ND	18 (69%)	ND	ND	ND	14 (70%)	ND	ND	67/95 (71%)
IAA	49 (100%)	ND	ND	ND	28 (72%)	6 (7%)	9 (45%)	31 (40%)	27 (93%)	150/312 (48%)
ACCD	5 (10%)	ND	ND	ND	31 (79%)	18 (20%)	14 (70%)	5 (5%)	3 (10%)	76/312 (24%)
Siderophore	32 (65%)	ND	ND	ND	29 (74%)	5 (5%)	12 (60%)	20 (24%)	13 (45%)	111/312 (54%)
Phosphate solubilization	36 (73%)	ND	ND	ND	24 (62%)	63 (69%)	10 (50%)	31 (40%)	5 (17)	169/312 (54%)
Nitrogen Fixation	16 (33%)	ND	ND	ND	ND	27 (30%)	4 (20%)	76 (90%)	5 (17%)	138/273 (73%)
Spore formation	ND	ND	19 (73%)	ND	ND	ND	ND	ND	ND	19/26 (73%)
Salinity tolerance (6% NaCl and higher)	33 (67%)	ND	ND	ND	ND	ND	ND	ND	ND	33/49 (67%)
Osmotic Tolerance (0.6 M sucrose)	49 (100%)	ND	ND	54 (69%)	ND	ND	ND	ND	ND	103/127 (81%)
Osmotic Tolerance (1.2 M sucrose)	47 (96%)	ND	ND	ND	ND	ND	ND	ND	ND	47/49 (96%)
Amylase	ND	ND	ND	10 (13%)	ND	ND	2 (10%)	ND	ND	12/98 (12%)
Host Plant	Rice	Rice	Rice	Rice	Lesdepeza sp.	Maize	Rice	Tomato	Marama bean	

ND – not determined

### Germination assay and early plant growth development

**Fig. 3:**
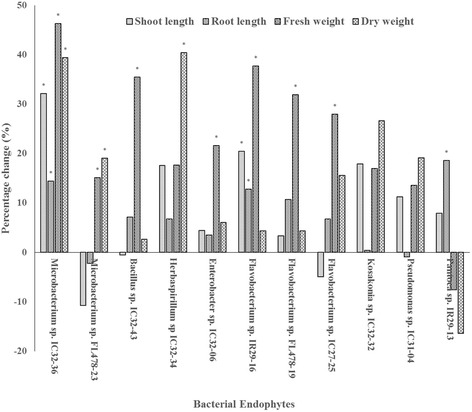
Percentage change of shoot length, root length, fresh weight and dry weight of IR29 seedling during early growth development after inoculation with selected endophytes. Asterisk (*) indicates statistically significant change over control at *P* ≤ 0.05 (*t*-test, SAS v9.4)

The effects of endophytic bacterial inoculation on rice seeds of the salt-sensitive cultivar IR29 were observed during germination under normal and 150 mM NaCl salt stress conditions as well as early seedling growth. Two strains, *Microbacterium* sp. IC37–36 and *Flavobacterium* sp. IR29–16 significantly and consistently increased shoot and root length and plant biomass. The detrimental effects of salt stress were observed on the germination rate and percentage of seeds, but inoculation with *Flavobacterium* sp*.* IC27–25, *Flavobacterium* sp. IC31–28 and *Xanthomonas* sp. IC31–27 significantly improved germination under salt stress conditions. *Flavobacterium* sp. strains isolated in the salt-sensitive cultivar were not able to significantly enhance germination parameters under salt stress conditions. Different rice cultivars contain an assemblage of bacterial endophytes that can promote germination and plant growth (Additional file 3: Figure S1a, b, c; Fig. 3).

### Endophytic adaptation of bacterial Endophytes from Rice seeds

Of the functional traits and characteristics studied which are putatively necessary for endophytic lifestyle of bacterial isolates, the activities of pectinase, cellulase and catalase were observed from more than 92% of the bacterial isolates, suggesting the importance of these traits to endophytic lifestyle. Motility and oxidase activity were also observed in 41% and 71% of the isolates, respectively. All of the bacterial isolates were also capable of surviving and even thriving in 0.6 M sucrose concentration while 96% were able to survive in 1.2 M sucrose concentration. Most of the isolates can tolerate an induced salt stress of 4% NaCl and higher. Only 3 isolates have the highest salt tolerance capable of growth in nutrient agar amended with 9% NaCl (Additional file 4: Table S2; Table 3).

### Distinguishing characteristics of Rice seed bacterial endophytes

**Fig. 4:**
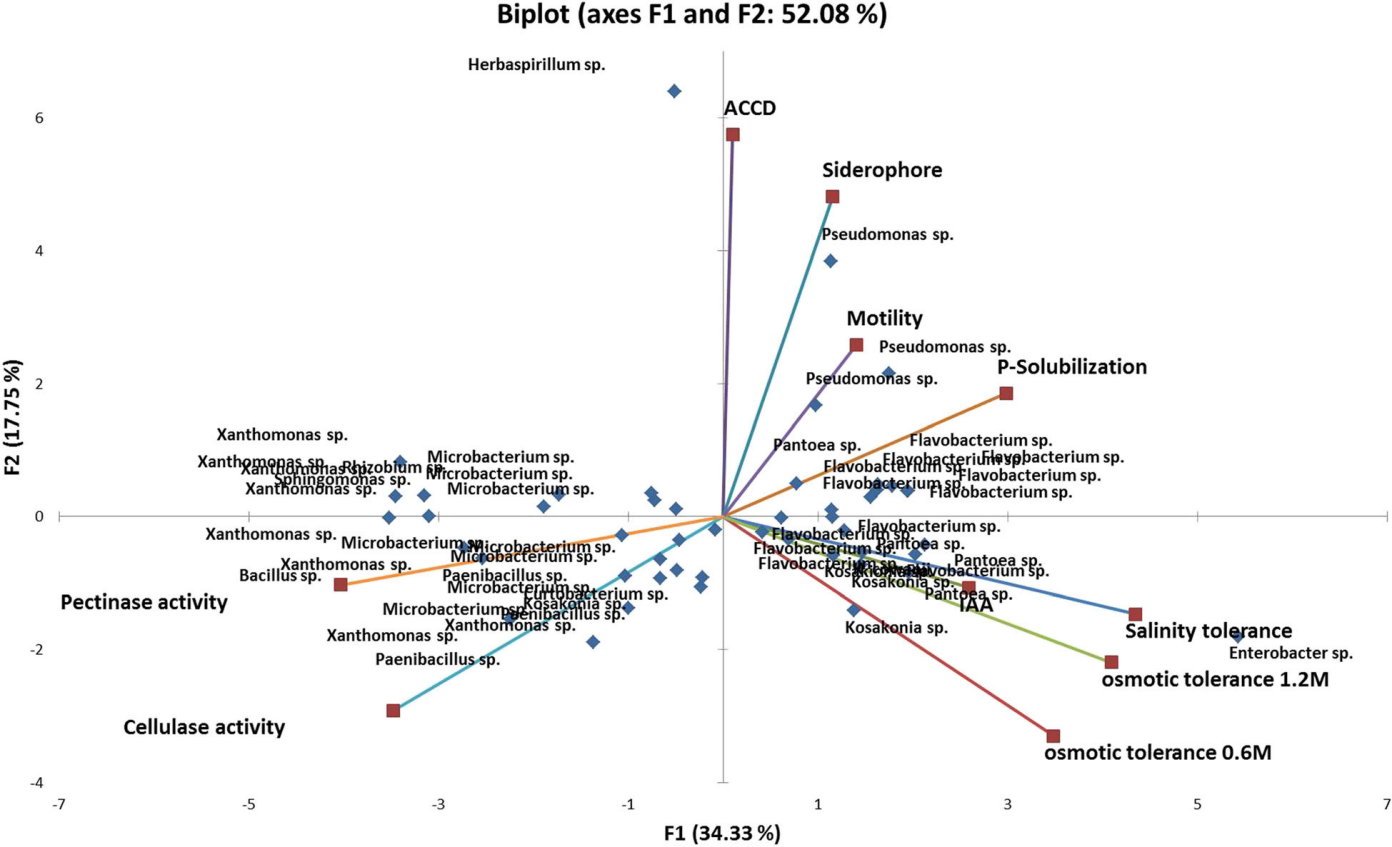
Bi-plot ordination diagram of principal component analysis describing plant growth promoting traits and functional activities of 49 bacterial endophytes isolated from salt-sensitive and salt-tolerant rice cultivars

Seed bacterial endophytes that belong to the same type strains possess similar PGP traits and physiological activities regardless of their isolation source, that is, whether they are isolated from the salt-sensitive or the salt-tolerant rice cultivar. Aside from the generally observed traits of seed endophytes, the bi-plot ordination of principal component analysis from 49 isolates reveals prominent distinguishing traits of several groups of rice seed bacterial endophytes. The ordination diagram using plant growth promoting traits (IAA, ACCD, phosphate solubilization) and physiological activities (salinity tolerance, osmotic tolerance and polymer-degrading enzymes) reveal three major groups occupying 3 quadrants (Fig. 4). *Microbacterium* sp. strains and *Xanthomonas* sp. strains isolated from the different rice cultivars are distinguished by their higher pectinase and cellulase activity. Siderophore production distinguishes different representatives of *Pseudomonas* strains. *Flavobacterium* sp. strains are characterized as better phosphate solubilizers and IAA producers with higher osmotic and salinity tolerance. Strains from *Kosakonia* and *Pantoea* are featured as IAA producing, salt and osmotic tolerant endophytes. There is a significantly positive correlation between bacterial pectinase and cellulase activity. A positive correlation also exists for the ability of bacterial endophytes to tolerate salt and osmotic stress. Interestingly, IAA is moderately correlated with osmotic tolerance at 1.2 M sucrose concentration.

Additional details in the PGP characters and physiological traits can also be observed in the different bacterial groups (Additional file 2: Table S1; Additional file 4: Table S2). *Microbacterium* sp. strains isolated in different rice cultivars do not produce siderophores and most do not have nitrogen fixing ability. *Microbacterium* sp. strains also tolerate to up to 4–6% salt concentration and 1.2 M sucrose concentration. They all have catalase activity, low cellulase activity and moderate pectinase activity. *Flavobacterium* sp. strains produce a higher degree of IAA in the presence of tryptophan from 10 to 20 μg ml^−1^, some are nitrogen fixing bacteria, produce siderophores and solubilize phosphorus. They all have catalase activity and low cellulase and pectinase activity. *Kosakonia* sp.*, Pantoea* spp. and *Xanthomonas* sp. strains generally produce smaller amounts of IAA ranging from 3 to 5 μg ml^−1^. They are mostly siderophore producers and phosphate solubilizers. *Xanthomonas* sp. strains show lower tolerance to salt stress where they can only survive up to 2% salt concentration. Osmotic tolerance varies where some strains have higher survival even at 1.2 M sucrose concentration. *Xanthomonas* sp. also shows very strong cellulase and pectinase activity. In this study, representatives of *Actinobacteria* and *Bacilli* do not produce siderophores.

## Discussion

This study investigated plant growth promotion and physiological characteristics related to the endophytic lifestyle of culturable bacterial endophytes inhabiting the seeds of rice. There are relatively diverse groups of culturable bacterial endophytes of the rice seeds (Fig. 1, Fig. 2, Additional file 1: Figure S2b). Characterization of seed endophytes in terms of growth promotion activity showed that each rice cultivar possessed a plethora of endophytes with multiple plant growth promotion abilities (Additional file 2: Table S1), some of which could significantly enhance germination rate, germination percentage and plant growth of the rice seeds during early development (Additional file 3: Figure S1a, b c, Fig. 3). Characterization of physiological and functional traits related to endophytic lifestyle particularly in the seeds showed that majority of the endophytes have putative endophytic adaptations (Table 3; Additional file 4: Table S2). On the other hand, several bacterial groups are distinguished by their PGP characters and physiological traits showing similar properties of strains that belong to the same bacterial type regardless of their isolation source (Fig. 4).

There are some bacterial groups such as *Flavobacterium*, *Microbacterium*, *Xanthomonas*, *Pantoea* and *Kosakonia* that were isolated in most of the rice cultivars. On the other hand, most of the isolated seed bacterial endophytes in this study were also observed in other studies on rice endophytes, though they were not isolated in all the cultivars studied, suggesting that these bacteria are widespread in the rice and that they are well adapted to the rice niche. *Flavobacterium* was detected in the leaves, roots and shoots [13, 36, 37]. *Xanthomonas* was detected in the leaves and seeds and acts as a biocontrol bacterium [36, 38]. *Microbacterium*, *Pantoea*, *Enterobacter* and *Herbaspirillum* were also commonly identified as endophytes in the rice host [6, 12, 13, 36, 37, 39, 40]. These major groups of bacteria were also seen as important members of the root endosphere [6]. Combining results of 16S rRNA and BOX PCR show that the closest reference strains of the isolated bacteria (*Microbacterium*, *Flavobacterium*, *Xanthomonas*, *Kosakonia*, *Paenibacillus*) from the seed potentially belong to the same bacterial species, but may have genetic differences up to the subspecies level as they form subclusters within their own groups [41, 42]. Many of the bacterial isolates also have high similarities with reference strains that were originally isolated as rice-associated bacteria (Additional file 5: Table S3). This study indicates that rice seeds act as a vector for transmission of these rice associated bacterial endophytes colonizing different parts of the rice plant and finally recolonizing the seeds. These seed endophytes are also present in many of the rice cultivars irrespective of the hosts’ genotype, physiological tolerance to salinity stress and phylogenetic relationship indicating that rice seeds are inhabited by potential core microbial communities found in other indica cultivars as well.

As bacterial endophytes colonize the plants, they may also be selectively maintained by their plant host due to their plant growth promoting traits or other beneficial properties. Rice seeds harbor an arsenal of bacterial endophytes with PGP traits that help during germination and early seedling development of seeds. Almost all of the ACCD bacteria isolated from the rice endosphere were able to promote growth during the early seedling development with *Microbacterium* sp. IC37–16 significantly enhancing germination and growth parameters. There were five ACCD positive isolates out of 49 in our study, but it seems to be a more prominent property of some endophytes as observed by other authors (Table 3). Beneficial bacteria containing ACCD aid in the development and plant growth by lowering ethylene levels primarily through cleaving ACC, the precursor of ethylene, to ammonia and α-ketobutyrate which consequently reduces the extent of ethylene growth inhibition [43, 44]. The advantageous effect of ACCD bacteria becomes prominent especially when the host plant experiences biotic or abiotic stress conditions. The process of deamination of ACC is a key plant growth promoting trait present in a wide range of bacteria such as *Azospirillum*, *Burkholderia* and *Agrobacterium* [45] and is essential for the promotion of growth and stress homeostasis regulation of the seedlings under saline conditions [46]. There is also an indication that ACCD is important during colonization as the production of ethylene is upregulated in plants during bacterial colonization especially by endophytes or pathogens [47]. Aside from ACCD, the phytohormone IAA is also a vital phytostimulator produced by bacteria. Indole-3-acetic acid, the most commonly produced auxin, is a signaling molecule in both plant and microorganisms essentially acting as a reciprocal signaling molecule in plant-microbe interaction [48]. This study showed that IAA production is a prominent feature of rice seed endophytes as well as other endophytes (Table 3). Most of the isolates in this study are capable of producing IAA in varying amounts. This observation was also reviewed by Kim et al. [49] showing that plants are colonized by high numbers of IAA-producing bacteria. Inoculation of plants with IAA producing PGPB causes changes in the root architecture stimulating root hair formation and increasing the number and length of lateral and primary roots ultimately increasing root surface area for mineral uptake and root exudation [50, 51]. *Flavobacterium* sp. IR29–16 and other *Flavobacterium* sp. strains could have promoted significant growth particularly in the root length of plants through IAA production. A wild-type strain *Pseudomonas putida* GR12–2 producing 32.7 μg mL^−1^ with 500 μg mL^−1^ tryptophan amendment while a mutant strain producing only 2.0 μg mL^−1^ IAA were still capable of increasing root length and formation of adventitious roots in canola seeds and mung bean, respectively [52]. Rice plant is also a host to many nitrogen-fixing endophytes isolated in the seeds, stems and roots [14] (Additional file 6: Figure S3). These diazotrophic bacteria, which are less likely to be found in soil, can also colonize the tissues of other gramineous plants forming small aggregates distributed in the plant body [53] yet does not necessarily form nodules [54]. Symbiotic biological nitrogen fixation between diazotrophic endophytes and tropical grasses including rice, show that some plants may obtain part of their nitrogen needs from such associations [55]. Siderophore production and phosphate solubilization may not have direct plant growth promotion effects during our study, but the fact that around 70% of the seed endophytes possess these traits suggest their importance during plant development. *Pseudomonas* strains from this study were distinguished by their high siderophore production. Fluorescent pseudomonas have been studied as capable of colonizing various ecological niches and this is reflected by the high diversity of their iron uptake systems [56]. Endophytes producing siderophore especially the *Pseudomonas* strains in this study have a selective advantage over other bacteria and pathogens since they overcome competing organisms by depriving them of iron [3, 8]. Rice plants were also observed to be colonized by consistent groups of siderophore-producing bacteria such as *Pantoea* in different plant parts and in different growth stages [57]. It has also been observed that some endophytes could migrate from the internal tissues to the external surface of the plants and even to the soil [58] and that several members of the seed endophytic community could colonize the rhizosphere and the surrounding soil [13]. During this time, phosphate solubilization may become important in the rhizosphere region of the roots.

Bacterial endophytes of rice seeds have prominent functional traits potentially important for their endophytic lifestyle and host adaptation [59]. Our study showed that majority of the seed endophytes are capable of producing cellulase (92%) and pectinase (96%) which was also observed by Elbetagy et al., [39] for rice endophytes. Some bacterial groups especially *Microbacterium* and *Xanthomonas* displayed higher secretion of cellulase and pectinase. Cellulase has been found to be widely distributed among *Xanthomonas* species and play a role in the degradation of plant cell walls [60]. Furthermore, plant-depolymerizing enzymes such as cellulase and pectinase are necessary during colonization and migration of endophytes from one location to another particularly in the degradation of the middle lamella [61] or through active penetration into the plants [39, 62, 63]. In *Burkholderia* sp. PsJN, cell wall-degrading endoglucanase, endopolygalacturonase and other associated enzymes were important in the entry into the root internal tissues of *Vitis vinifera* [64]. The same bacterial strain was also able to colonize the interior of young berries as well as the inflorescence organs such as grape stalks and immature berries primarily through the xylem vessels [65]. In addition, motility of bacteria may also be required for adhesion on plant surfaces [6]. In this study, motility is observed in 41% of the isolates, but was observed at 100% occurrence in other studies for rice endophytes [4, 39]. Kaga et al. [12] also showed that motility (74% of their isolate) is more prominent in rice endophytes isolated in the shoots and phyllosphere compared to those in the roots, indicating the importance of motility in colonization and migration. Hardoim et al. [13] and Mano et al. [66] found that the seed endophytic community is more similar to the shoot community compared to the roots indicating that seed-borne endophytes rapidly and actively migrate from the spermosphere to the shoot endosphere. Such ability generally requires the production of plant-polymer degrading enzymes and motility or some sort for translocation. Catalase which helps detoxify ROS, can also be an essential component for successful survival of the colonizing endophyte during oxidative burst by plants [67]. Reactive oxygen species (ROS) are usually produced during endophytic colonization and pathogen invasion as a typical plant defense response. ROS-scavenging enzymes were also important in the endophytic colonization in the roots of rice as a means of counteracting the accumulation of ROS [68]. Catalase along with other ROS-scavenging enzymes was also found to be upregulated by *Gluconacetobacter diazotrophicus* during colonization and interactions with sugarcane following oxidative stress by the host plant [69]. Okunishi et al. [4] and Hardoim et al. [70] showed that catalase activity is present in many endophytes colonizing the rice plant as also observed in this study. The seed as an exclusive microhabitat may also require additional adaptation as it imposes a desiccating environment particularly in the mature seeds. In addition, during germination of seeds, osmotic pressure increases with the release of oligosaccharides and sugars necessitating the need for adaptation of seed endophytes to high osmotic pressure [12]. Furthermore, most of the isolates in this study can tolerate high osmotic stress which also points out that the seed can be a selective microhabitat to bacteria that can survive in the seed endosphere during seed maturation and seed germination. *Flavobacterium*, *Kosakonia* and *Pantoea* strains are notably salt tolerant and osmotic tolerant seed bacteria as presented in the results. There is also a significantly positive correlation between bacterial salt and osmotic tolerance. Surprisingly, the same bacterial strains capable of surviving in high osmotic stress can also tolerate high salinity conditions indicating dual adaptation as osmotic and salinity stress inflict the same initial limiting conditions [71–73] and these bacteria may have evolved the same mechanisms to combat these abiotic stresses. The almost ubiquitous activity of the polymer-degrading enzymes cellulase and pectinase as well as the ability to detoxify ROS through catalase by bacterial endophytes conform with metagenomic studies showing that these physiological characters are vital for endophytic competence. Comparative metagenomic analysis between endophytes, phytopathogens, rhizosphere bacteria and soil bacteria together with metagenomic analysis of the rice roots show that endophytes possess prominent features and metabolic processes important, if not required, for endophytic lifestyle including flagella or motility mechanisms, plant-polymer degrading enzymes, iron sequestration and storage and scavenging or detoxification of reactive oxygen species [6, 7].

## Conclusions

The findings of the study not only support the use of seed bacterial endophytes as potential plant growth promoters but also give an overview of the general physiological features important for endophytic lifestyle, especially in the seeds. The study points out that the rice seed may selectively screen endophytes with physiological characteristics associated with tolerance to osmotic stress, ability to detoxify ROS and production of plant polymer-degrading enzymes. The study showed that rice seed bacterial endophytes possess physiological traits important for their survival in the seed endosphere along with plant growth promoting potential that enables them to modulate rice plant growth during germination and early seedling development. This study established that the seeds of rice plants are inhabited by several groups of bacteria isolated from many of the indica cultivars irrespective of the hosts’ genotype, physiological adaptation to salt stress and phylogenetic lineages indicating the presence of core microbial communities transmitted via the seeds as they become common endophytes associated with rice. These groups also manifest prominent traits that distinguish them from other groups of bacteria and indicate key determinants for their success as endophytes in the seed microbiome. Further mechanisms of plant growth promotion as well as complete endophytic characterization and their responses to survival, colonization and maintenance in the plant host still need to be addressed to understand complex plant-biotic relationships.

## Additional files

Additional file 1: Figure S2. **a** Photograph of BOX PCR genomic fingerprints of rice seed endophytes resolved on an ethidium bromide stained agarose gel. 01, 14, 30, 31, 46 and 55: DNA ladder, 02–13: *Flavobacterium* sp. (IC27–01, IC31–02, IC31–03, IC32–07, IR29–16, IR29–17, FL478–19, FL478–21, IC27–25, IC31–28, IC32–33IC37–35,), 15–18: *Pantoea* sp. (IR29–13, IR29–15, FL478–22, IC31–29), 18–23: *Kosakonia* sp. (IC32–10, IC32–12, IC27–24, IC32–32, IC32–46), 24–26: *Paenibacillus* sp. (FL478–18, IC32–31, IC32–42), 27–29: *Pseudomonas* sp. (IC32–08, IC31–04, IC32–09), 32–39: *Xanthomonas* sp. (IC31–05, IC32–11, IR29–14, FL478–20, IC31–27, IC32–30,IC31–44, IR29–49), 40: *Enterobacter* sp. 34, 41: *Herbaspririllum* sp. 34, 42: *Rhizobium* sp. 47, 43: *Sphingomonas* sp. 26, 44: *Bacillus* sp. 43, 45: *Curtobacterium* sp. 37, 47–54: *Microbacterium* sp. (IC37–36, FL478–23, IC37–38,IC37–39, IC37–40, IC37–41, IC31–45, IR29–48). **b** Cluster analysis of BOX PCR genomic fingerprints of 49 endophytic bacteria isolated from the seed endosphere of indica rice cultivars. The dendrogram was constructed using SPSS Statistics Version 20 using heirarchichal cluster analysis. (ZIP 210 kb)

Additional file 2: Table S1. PGP traits of bacterial endophytes in rice seeds. (DOCX 66 kb)

Additional file 3: Figure S1. **a** Percentage change in germination parameters of IR29 seeds after inoculation with endophytes from *Actinobacteria*, *Bacilli*, α- and β-*Proteobacteria*. **b** Percentage change in germination parameters of IR29 seeds after inoculation with endophytes from *Flavobacterium* and *Kosakonia*. **c** Percentage change in germination parameters of IR29 seeds after inoculation with endophytes from *Pantoea*, *Pseudomonas* and *Xanthomonas. (ZIP 262 kb)*

Additional file 4: Table S2. Functional traits and metabolic activity associated to endophytic adaptation of bacterial endophytes in seeds of rice. (DOCX 25 kb)

Additional file 5: Table S3. Information on the seed bacterial endophytes isolated from the different rice cultivars showing accession number, closest type strain and closest rice associated bacteria. (XLSX 39 kb)

Additional file 6: Figure S3. Amplification of the *nif*H gene of seed bacterial endophytes showing nested PCR product with ~317 bp fragment as amplified with nifHFor and nifHRev primer set. (JPEG 71 kb)

## Abbreviations

ACC: 1-aminocyclopropane-1-carboxylate
ACCD: 1-aminocyclopropane-1-carboxylate deaminase
ANOVA: Analysis of Variance
CAS: Chrome azurol S medium
CFU: Colony forming unit
CMC: Carboxymethylcellulose
dS m^−1^: DeciSiemens per meter
FL478: Moderately salt-tolerant cultivar; IR669646-3R-178-1-1
IAA: Indole-3-acetic acid
IC27: Moderately salt-tolerant cultivar; CSR 28
IC31: Moderately salt-tolerant cultivar; IR55179-3B-11–3
IC32: Moderately salt-tolerant cultivar; IR58443-6B-10-3
IC37: Highly salt-tolerant cultivar; IRRI154
IR29: Salt sensitive rice cultivar
IRRI: International Rice Research Institute, Philippines
LSD: Least significant difference
NBRIP-BPB: National Botanical Research Institute Phosphate-bromophenol blue medium
OD: Optical density
PCA: Principal component analysis
PCR: Polymerase chain reaction
PGP: Plant growth promotion
PGPR: Plant growth promoting rhizobacteria
R2A: Reasoner’s 2A medium
RDA: Rural Development Administration, South Korea
ROS: Reactive oxygen species
rpm: Revolutions per minute
α-KB: Alpha ketobutyrate
