# Supplementary figures and images for: Characterizing endophytic competence and plant growth promotion of bacterial endophytes inhabiting the seed endosphere of Rice
Source: BMC Microbiol. 2017 Oct 26;17:209. doi: 10.1186/s12866-017-1117-0 (PMC5658939; doi:10.1186/s12866-017-1117-0)

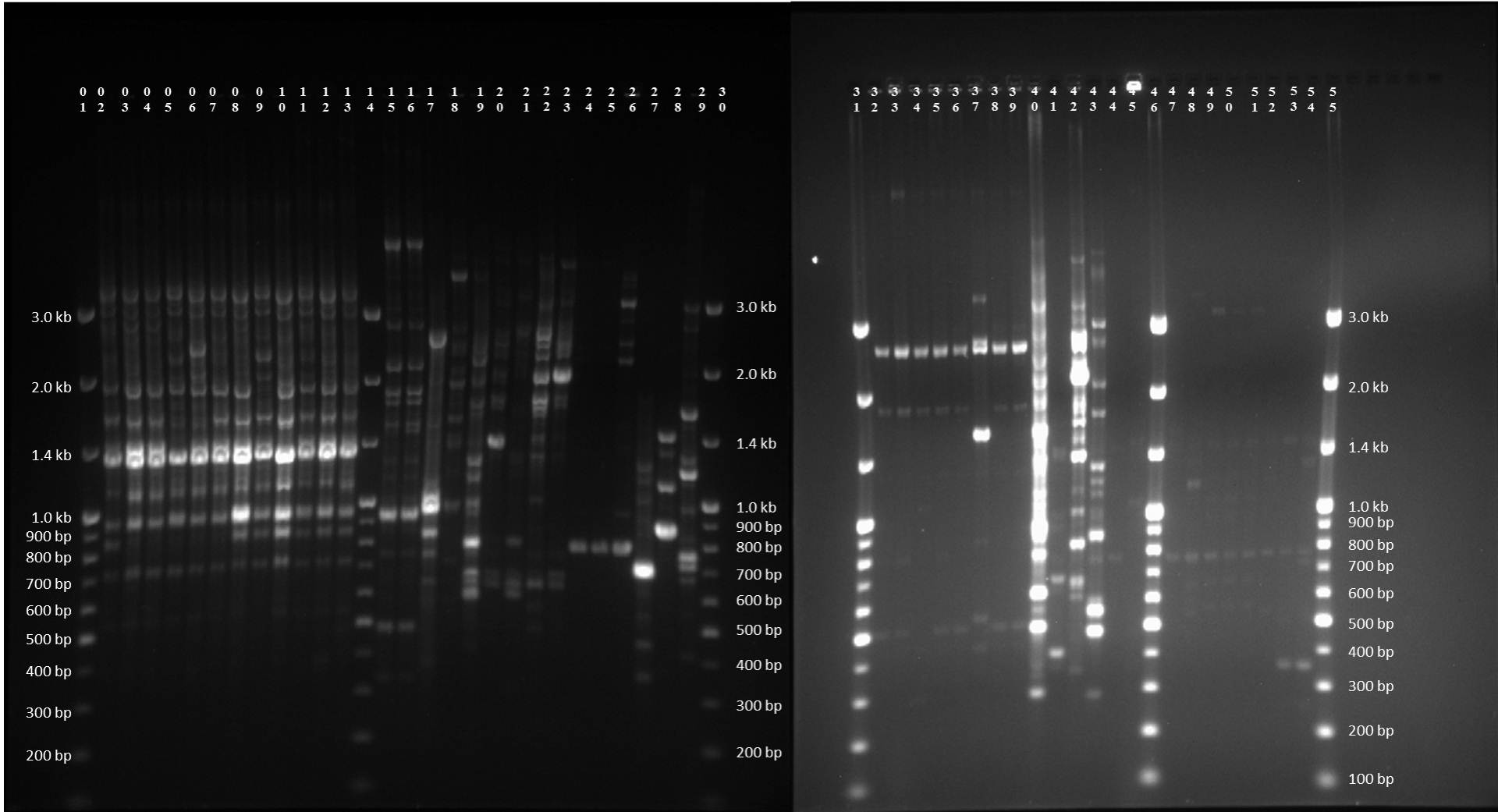

Supplement: Supplementary file 1 — a Photograph of BOX PCR genomic fingerprints of rice seed endophytes resolved on an ethidium bromide stained agarose gel. 01, 14, 30, 31, 46 and 55: DNA ladder, 02–13: Flavobacterium sp. (IC27–01, IC31–02, IC31–03, IC32–07, IR29–16, IR29–17, FL478–19, FL478–21, IC27–25, IC31–28, IC32–33IC37–35,), 15–18: Pantoea sp. (IR29–13, IR29–15, FL478–22, IC31–29), 18–23: Kosakonia sp. (IC32–10, IC32–12, IC27–24, IC32–32, IC32–46), 24–26: Paenibacillus sp. (FL478–18, IC32–31, IC32–42), 27–29: Pseudomonas sp. (IC32–08, IC31–04, IC32–09), 32–39: Xanthomonas sp. (IC31–05, IC32–11, IR29–14, FL478–20, IC31–27, IC32–30,IC31–44, IR29–49), 40: Enterobacter sp. 34, 41: Herbaspririllum sp. 34, 42: Rhizobium sp. 47, 43: Sphingomonas sp. 26, 44: Bacillus sp. 43, 45: Curtobacterium sp. 37, 47–54: Microbacterium sp. (IC37–36, FL478–23, IC37–38,IC37–39, IC37–40, IC37–41, IC31–45, IR29–48). b Cluster analysis of BOX PCR genomic fingerprints of 49 endophytic bacteria isolated from the seed endosphere of indica rice cultivars. The dendrogram was constructed using SPSS Statistics Version 20 using heirarchichal cluster analysis. (ZIP 210 kb) [file 12866_2017_1117_MOESM1_ESM.zip › S2a Figure BOX PCR.jpg]

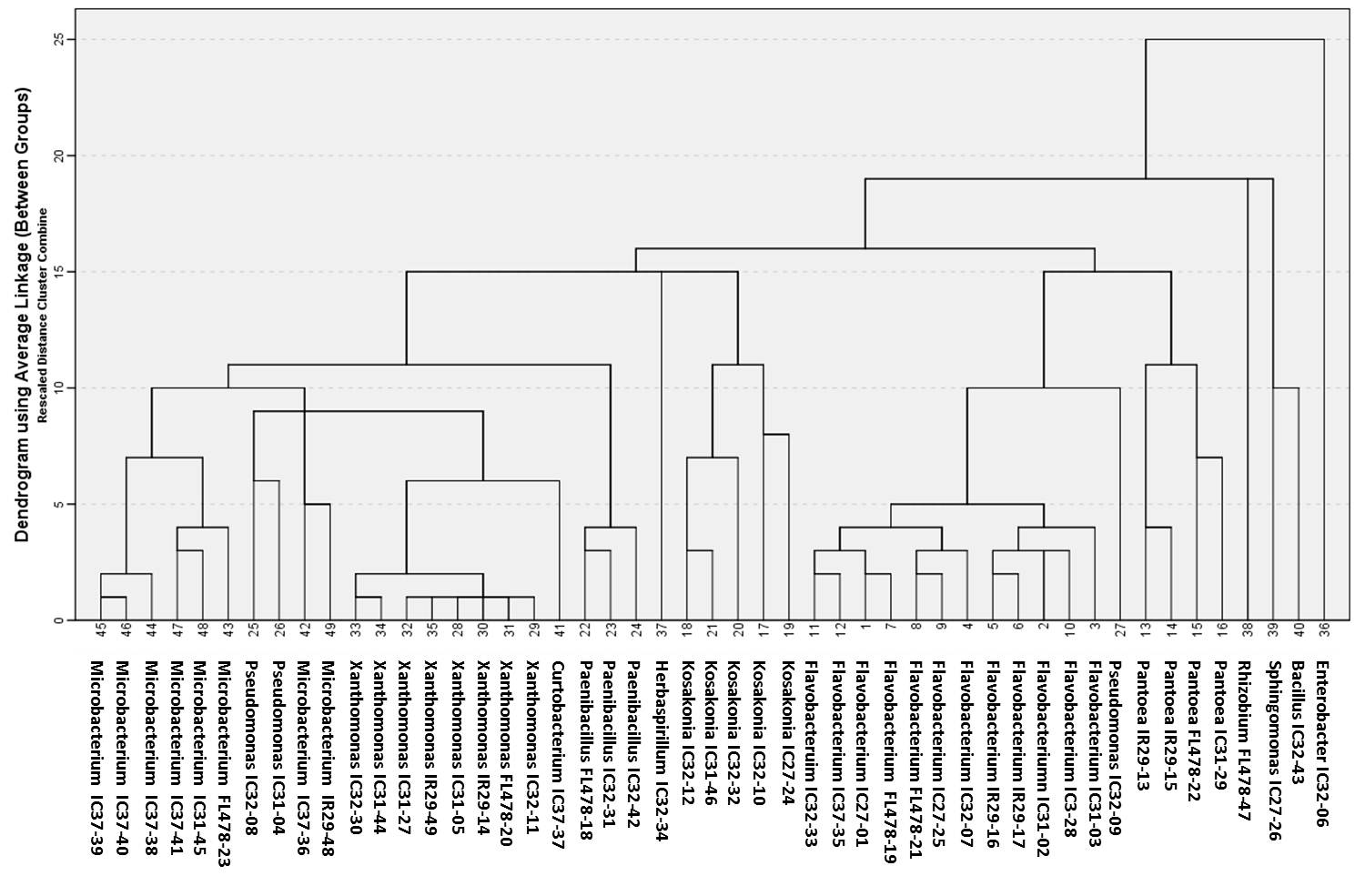

Supplement: Supplementary file 1 — a Photograph of BOX PCR genomic fingerprints of rice seed endophytes resolved on an ethidium bromide stained agarose gel. 01, 14, 30, 31, 46 and 55: DNA ladder, 02–13: Flavobacterium sp. (IC27–01, IC31–02, IC31–03, IC32–07, IR29–16, IR29–17, FL478–19, FL478–21, IC27–25, IC31–28, IC32–33IC37–35,), 15–18: Pantoea sp. (IR29–13, IR29–15, FL478–22, IC31–29), 18–23: Kosakonia sp. (IC32–10, IC32–12, IC27–24, IC32–32, IC32–46), 24–26: Paenibacillus sp. (FL478–18, IC32–31, IC32–42), 27–29: Pseudomonas sp. (IC32–08, IC31–04, IC32–09), 32–39: Xanthomonas sp. (IC31–05, IC32–11, IR29–14, FL478–20, IC31–27, IC32–30,IC31–44, IR29–49), 40: Enterobacter sp. 34, 41: Herbaspririllum sp. 34, 42: Rhizobium sp. 47, 43: Sphingomonas sp. 26, 44: Bacillus sp. 43, 45: Curtobacterium sp. 37, 47–54: Microbacterium sp. (IC37–36, FL478–23, IC37–38,IC37–39, IC37–40, IC37–41, IC31–45, IR29–48). b Cluster analysis of BOX PCR genomic fingerprints of 49 endophytic bacteria isolated from the seed endosphere of indica rice cultivars. The dendrogram was constructed using SPSS Statistics Version 20 using heirarchichal cluster analysis. (ZIP 210 kb) [file 12866_2017_1117_MOESM1_ESM.zip › S2b Figure BOX PCR.jpg]

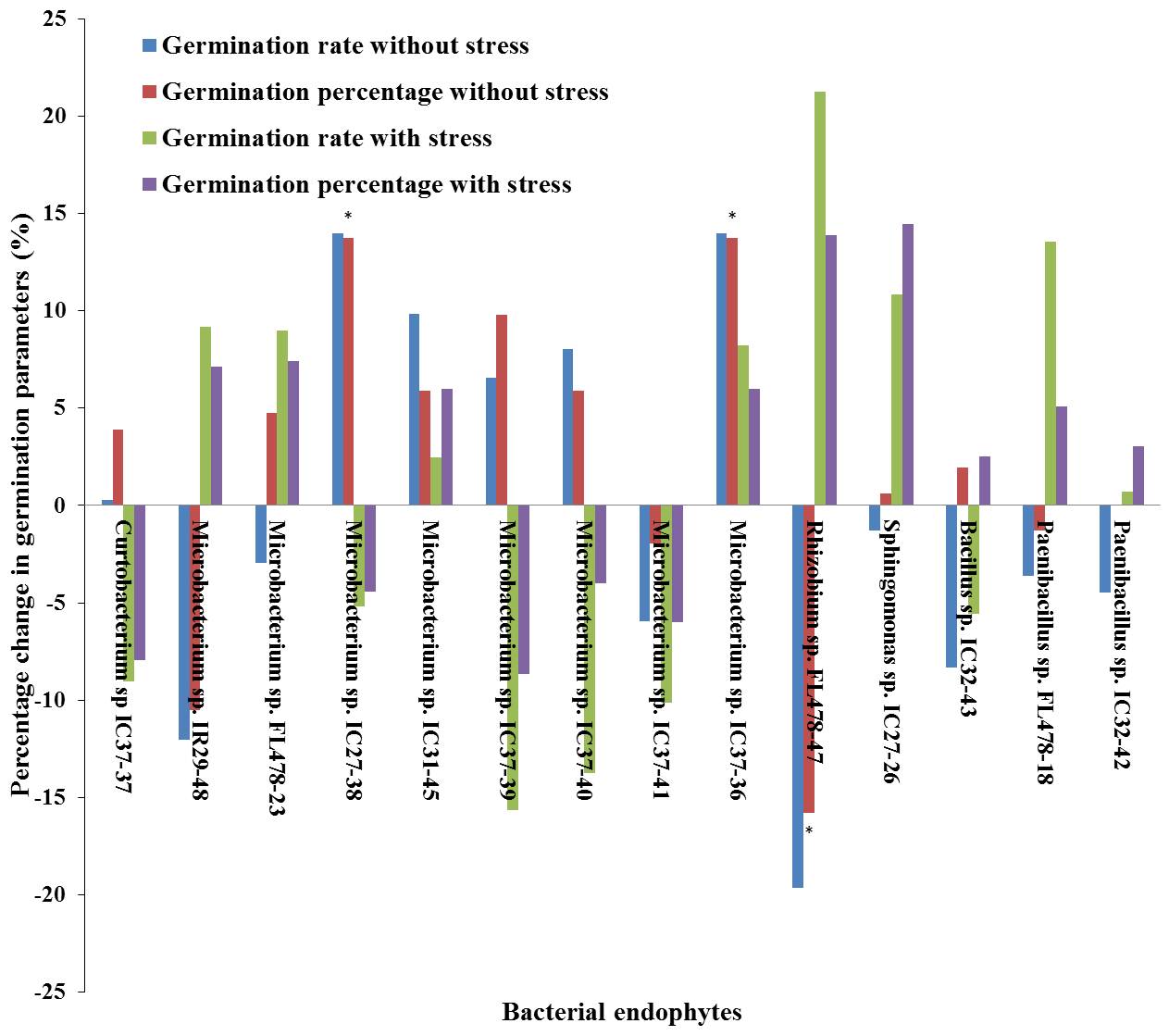

Supplement: Supplementary file 3 — a Percentage change in germination parameters of IR29 seeds after inoculation with endophytes from Actinobacteria, Bacilli, α- and β-Proteobacteria. b Percentage change in germination parameters of IR29 seeds after inoculation with endophytes from Flavobacterium and Kosakonia. c Percentage change in germination parameters of IR29 seeds after inoculation with endophytes from Pantoea, Pseudomonas and Xanthomonas. (ZIP 262 kb) [file 12866_2017_1117_MOESM3_ESM.zip › S1a_Figure.germination.jpg]

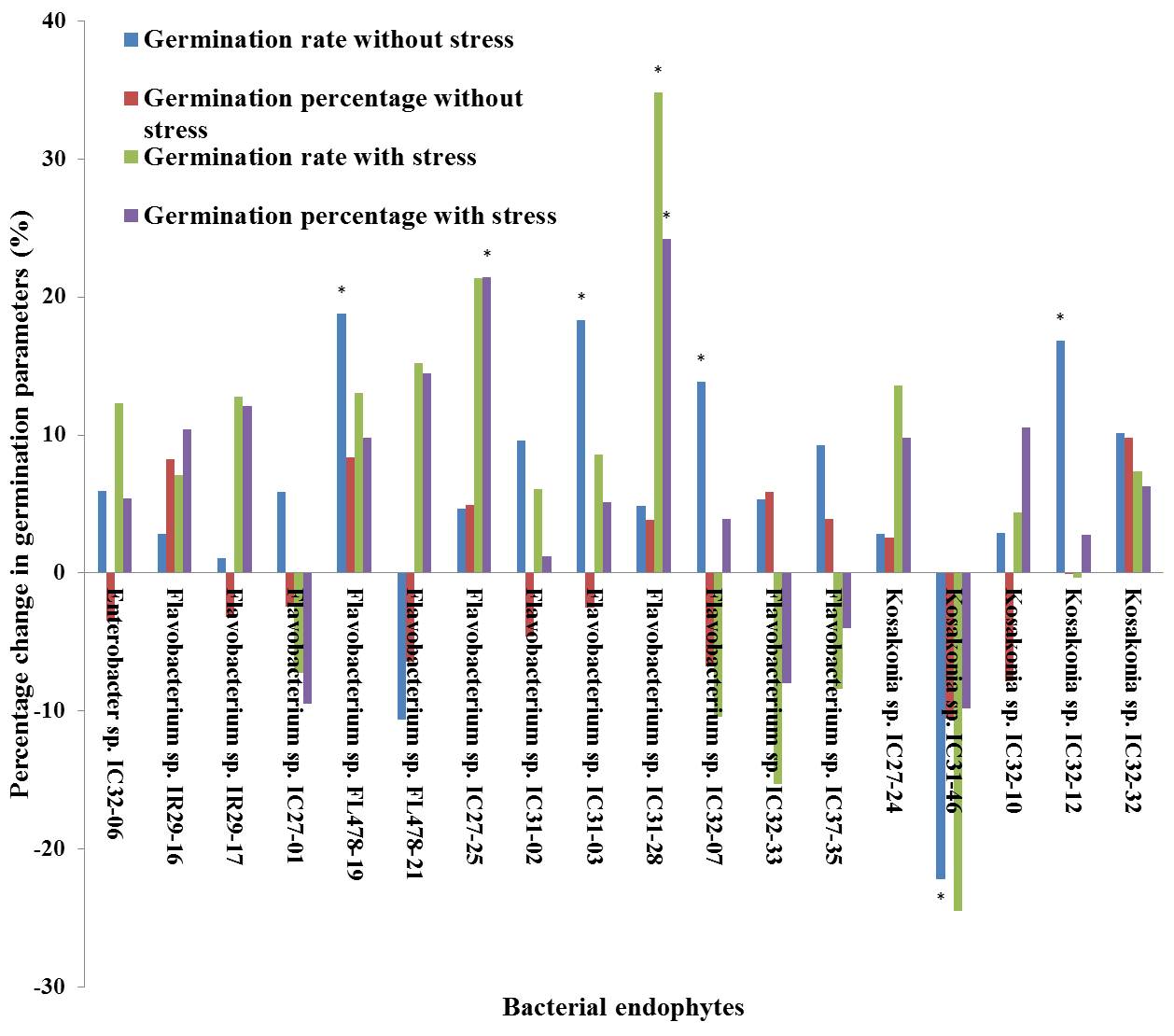

Supplement: Supplementary file 3 — a Percentage change in germination parameters of IR29 seeds after inoculation with endophytes from Actinobacteria, Bacilli, α- and β-Proteobacteria. b Percentage change in germination parameters of IR29 seeds after inoculation with endophytes from Flavobacterium and Kosakonia. c Percentage change in germination parameters of IR29 seeds after inoculation with endophytes from Pantoea, Pseudomonas and Xanthomonas. (ZIP 262 kb) [file 12866_2017_1117_MOESM3_ESM.zip › S1b_Figure.germination.jpg]

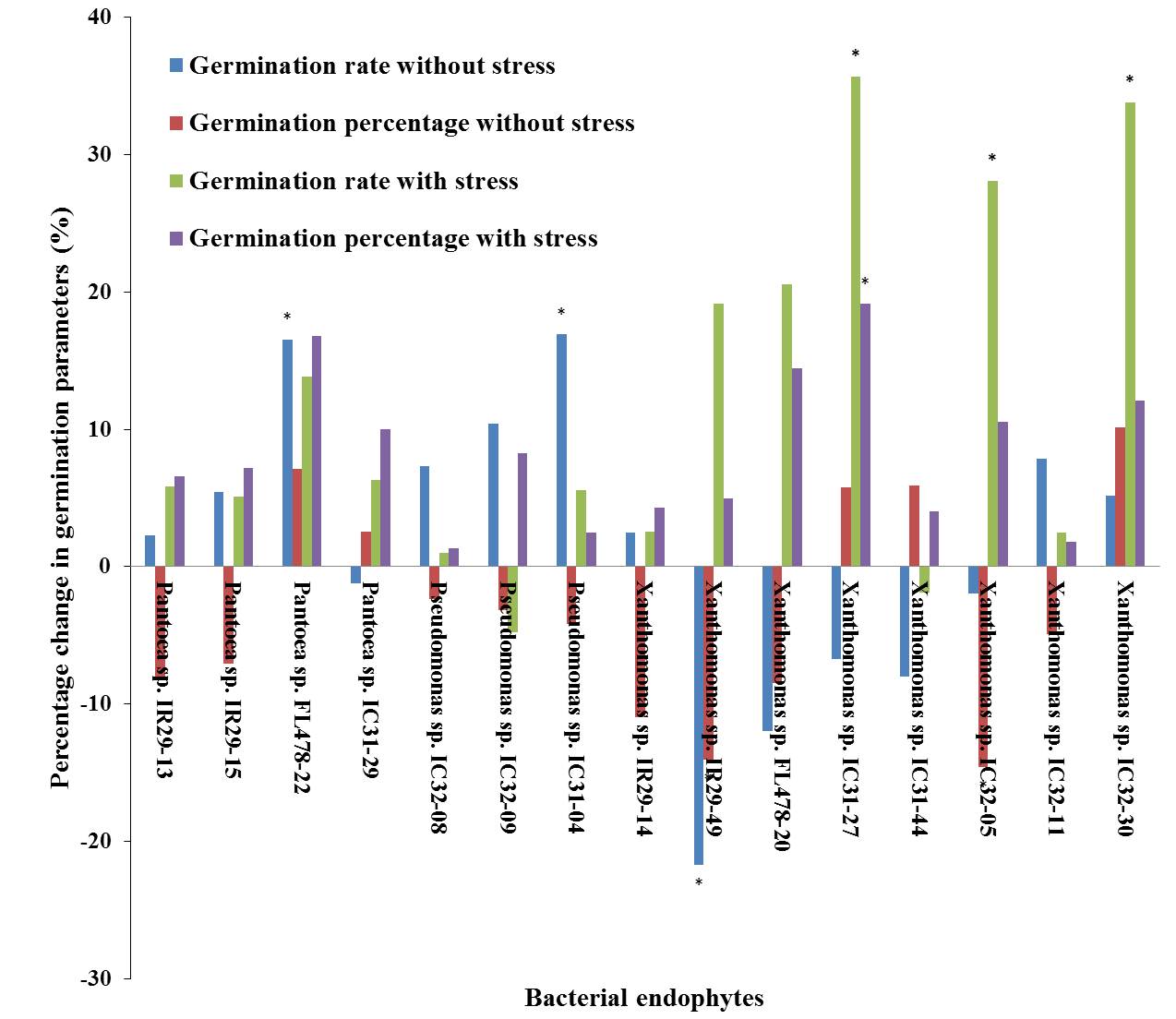

Supplement: Supplementary file 3 — a Percentage change in germination parameters of IR29 seeds after inoculation with endophytes from Actinobacteria, Bacilli, α- and β-Proteobacteria. b Percentage change in germination parameters of IR29 seeds after inoculation with endophytes from Flavobacterium and Kosakonia. c Percentage change in germination parameters of IR29 seeds after inoculation with endophytes from Pantoea, Pseudomonas and Xanthomonas. (ZIP 262 kb) [file 12866_2017_1117_MOESM3_ESM.zip › S1c_Figure.germination.jpg]

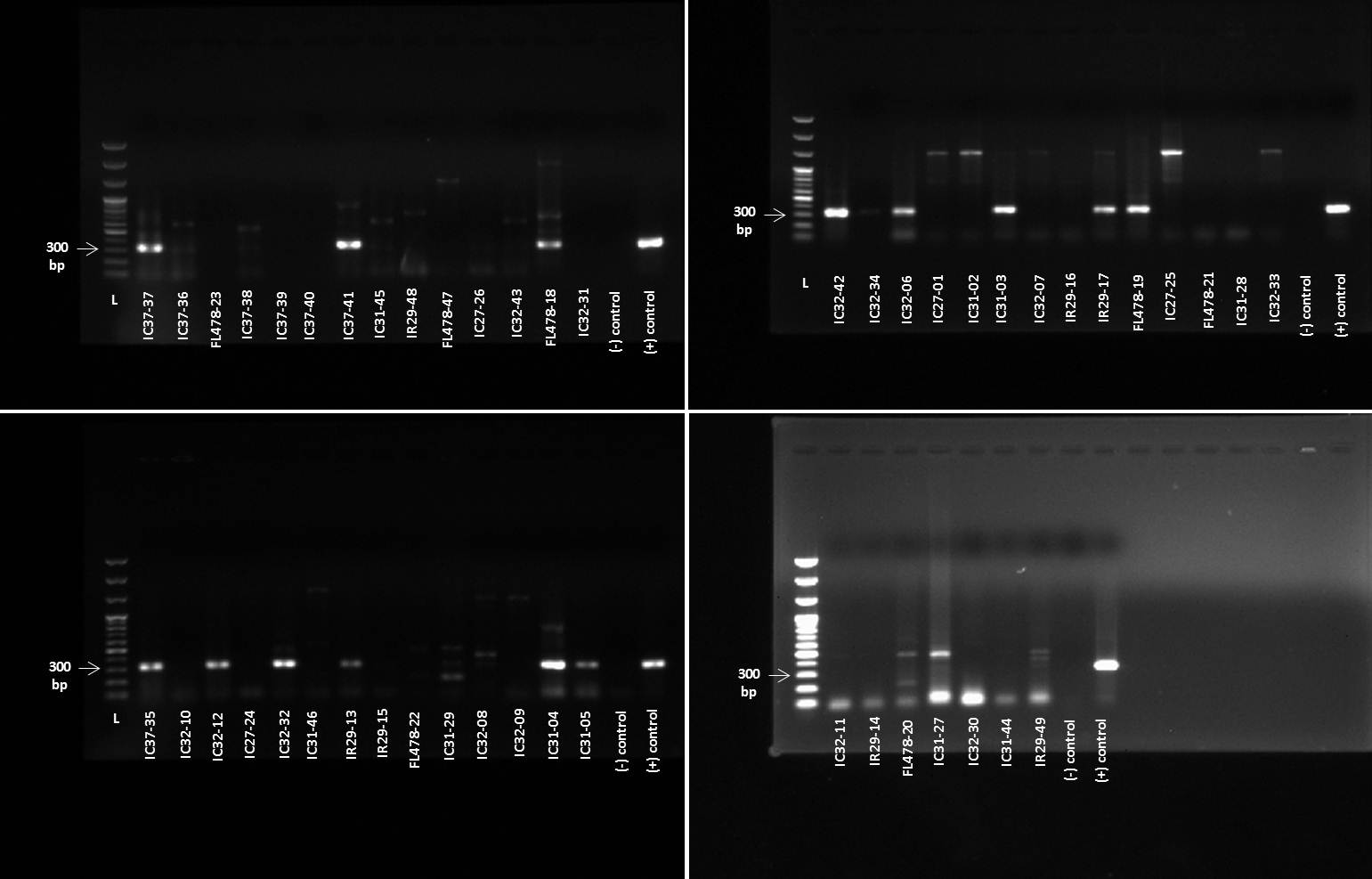

Supplement: Supplementary file 6 — Amplification of the nifH gene of seed bacterial endophytes showing nested PCR product with ~317 bp fragment as amplified with nifHFor and nifHRev primer set. (JPEG 71 kb) [file 12866_2017_1117_MOESM6_ESM.jpg]
