# Supplementary material for: Characterizing endophytic competence and plant growth promotion of bacterial endophytes inhabiting the seed endosphere of Rice
Source: BMC Microbiol. 2017 Oct 26;17:209. doi: 10.1186/s12866-017-1117-0 (PMC5658939; doi:10.1186/s12866-017-1117-0)
Supplement: Supplementary file 2 — PGP traits of bacterial endophytes in rice seeds. (DOCX 66 kb) [file 12866_2017_1117_MOESM2_ESM.docx]

Supplementary Data

S1 Table. PGP traits of bacterial endophytes in rice seeds

| Strain | IAA^a^ | | ACCD^b^ | N^c^ | S^d^ | P^e^ |
| --- | --- | --- | --- | --- | --- | --- |
|  | trp (+) | trp (-) |  |  |  |  |
| *Curtobacterium citreum* IC37-37 | 4.8±0.1 | 3.0±0.2 | - | + | ND | + |
| *Microbacterium hydrothermale* IC37-36 | 15.5±0.7 | 3.5±0.2 | 470.2853 | - | ND | + |
| *Microbacterium testaceum* FL478-23 | 10.3±0.1 | 1.8±0.04 | - | - | ND | + |
| *Microbacterium testaceum* IC37-38 | 5.7±0.2 | 2.0±0.2 | - | - | ND | + |
| *Microbacterium testaceum* IC37-39 | 6.6±0.1 | 2.2±0.1 | - | - | ND | + |
| *Microbacterium testaceum* IC37-40 | 6.6±0.2 | 2.1±0.2 | - | - | ND | + |
| *Microbacterium testaceum* IC37-41 | 9.7±0.8 | 2.2±0.02 | - | + | ND | + |
| *Microbacterium testaceum* IC31-45 | 14.5±0.1 | 3.9±0.3 | - | - | ND | - |
| *Microbacterium testaceum* IR29-48 | 11.5±0.4 | 3.1±0.1 | - | - | ND | - |
| *Rhizobium larrymoorei* FL478-47 | 24.4±0.8 | 6.5±0.3 | - | - | - | + |
| *Sphingomonas pseudosanguinis* IC27-26 | 25.9±1.6 | 2.4±0.1 | - | - | ND | + |
| *Bacillus thuringensis* IC32-43 | 2.4±0.03 | 1.94±0.1 | - | - | ND | + |
| *Paenibacillus hunanensis* FL478-18 | 14.9±0.8 | 2.5±0.2 | 1602.675 | + | ND | + |
| *Paenibacillus hunanensis* IC32-31 | 8.3±0.3 | 2.0±0.1 | - | - | ND | - |
| *Paenibacillus hunanensis* IC32-42 | 11.5±0.3 | 2.0±0.05 | - | + | ND | + |
| *Herbaspirillum huttiense* IC32-34 | 4.2±0.3 | 1.8±0.2 | 7036.203 | + | + | + |
| *Enterobacter* sp. IC32-06 | 66.0±1.3 | 30.3±0.8 | - | + | + | - |
| *Flavobacterium acidificum* IC27-01 | 13.8±0.4 | 4.3±0.2 | - | - | + | + |
| *Flavobacterium acidificum* IC31-02 | 20.0±1.3 | 4.2±0.1 | - | - | + | + |
| *Flavobacterium acidificum* IC31-03 | 18.7±0.7 | 4.1±0.02 | - | + | + | + |
| *Flavobacterium acidificum* IC32-07 | 18.1±0.6 | 4.8±0.05 | - | - | + | + |
| *Flavobacterium acidificum* IR29-16 | 14.1±0.1 | 4.7±0.1 | - | - | + | + |
| *Flavobacterium acidificum* IR29-17 | 17.5±0.4 | 5.0±0.1 | - | + | + | + |
| *Flavobacterium acidificum* FL478-19 | 13.9±0.4 | 4.2±0.03 | - | + | + | + |
| *Flavobacterium acidificum* IC27-25 | 14.4±0.4 | 4.3±0.02 | - | - | + | + |

^a^ μg/mL; ^b^ nmol a-KB h^-1^ mg protein^-1^; ^c^ Nitrogen fixation as assessed with amplification of *nif*H gene; ^d^ Siderophore production; ^e^ Phosphate solubilization; ND (not determined).

Continue

| Strain | IAA^a^ | | ACCD^b^ | N^c^ | S^d^ | P^e^ |
| --- | --- | --- | --- | --- | --- | --- |
|  | trp (+) | trp (-) |  |  |  |  |
| *Flavobacterium acidificum* FL478-21 | 18.3±0.2 | 4.1±0.04 | - | - | + | - |
| *Flavobacterium acidificum* IC31-28 | 13.2±0.3 | 3.2±0.04 | - | - | + | - |
| *Flavobacterium acidificum* IC32-33 | 11.9±0.05 | 2.5±0.1 | - | - | + | + |
| *Flavobacterium acidificum* IC37-35 | 9.7±0.06 | 2.9±0.5 | - | + | + | - |
| *Kosakonia cowanii* IC32-10 | 4.2±0.7 | 1.4±0.04 | - | - | + | + |
| *Kosakonia cowanii* IC32-12 | 3.4±0.1 | 1.4±0.1 | - | + | - | - |
| *Kosakonia cowanii* IC27-24 | 3.05±0.2 | 1.6±0.1 | - | - | + | + |
| *Kosakonia cowanii* IC32-32 | 3.5±0.1 | 1.5±0.1 | 498.5278 | + | + | + |
| *Kosakonia cowanii* IC31-46 | 4.9±1.1 | 1.4±0.02 | - | - | + | + |
| *Pantoea* sp. IR29-13 | 7.9±0.5 | 1.3±0.02 | - | + | - | + |
| *Pantoea* sp. IR29-15 | 18.4±2.1 | 2.7±0.2 | - | - | + | - |
| *Pantoea dispersa* FL478-22 | 7.3±0.6 | 1.98±0.05 | - | - | + | + |
| *Pantoea dispersa* IC31-29 | 4.5±0.7 | 1.3±0.01 | - | - | + | + |
| *Pseudomonas argentinensis* IC32-08 | 3.3±0.2 | 3.5±0.5 | - | + | + | + |
| *Pseudomonas parafulva IC32*-09 | 3.2±0.3 | 2.1±0.03 | - | - | + | - |
| *Pseudomonas oryzihabitans* IC31-04 | 16.9±0.6 | 3.8±0.1 | 5457.813 | + | + | + |
| *Xanthomonas sacchari* IC31-05 | 4.5±0.5 | 2.0±0.1 | - | + | + | + |
| *Xanthomonas sacchari* IC32-11 | 5.5±0.8 | 2.1±0.1 | - | - | + | + |
| *Xanthomonas sacchar*i IR29-14 | 4.1±0.4 | 2.7±0.1 | - | - | + | + |
| *Xanthomonas sacchari* FL478-20 | 5.3±0.06 | 3.4±0.1 | - | - | + | + |
| *Xanthomonas sacchari* IC31-27 | 3.9±0.4 | 2.2±0.1 | - | - | + | - |
| *Xanthomonas sacchari* IC32-30 | 5.3±0.3 | 2.2±0.2 | - | - | + | + |
| *Xanthomonas sacchari* IC31-44 | 4.1±0.1 | 1.62±0.1 | - | - | + | - |
| *Xanthomonas sacchari* IR29-49 | 2.8±0.6 | 1.5±0.1 | - | - | + | + |

^a^ μg/mL; ^b^ nmol a-KB h^-1^ mg protein^-1^; ^c^ Nitrogen fixation as assessed with amplification of *nif*H gene; ^d^ Siderophore production; ^e^ Phosphate solubilization; (+) positive; (-) negative; ND (not determined).
