# Supplementary material for: Characterizing endophytic competence and plant growth promotion of bacterial endophytes inhabiting the seed endosphere of Rice
Source: BMC Microbiol. 2017 Oct 26;17:209. doi: 10.1186/s12866-017-1117-0 (PMC5658939; doi:10.1186/s12866-017-1117-0)
Supplement: Supplementary file 4 — Functional traits and metabolic activity associated to endophytic adaptation of bacterial endophytes in seeds of rice. (DOCX 25 kb) [file 12866_2017_1117_MOESM4_ESM.docx]

Supplementary Data: S2 Table. Functional traits and metabolic activity associated to endophytic adaptation of bacterial endophytes in seeds of rice

| **Strain** | **Salinity tolerance (%)** | **Osmotic tolerance 0.6 M sucrose** | **Osmotic tolerance 1.2 M sucrose** | **Oxidase** | **Catalase** | **Motility** | **Cellulase** | **Pectinase** |
| --- | --- | --- | --- | --- | --- | --- | --- | --- |
| *Curtobacterium citreum* IC37-37 | 8 | 1.710 | 0.455 | + | + | - | + | ++ |
| *Microbacterium hydrothermale* IC37-36 | 6 | 1.082 | 0.568 | + | + | - | + | + |
| *Microbacterium testaceum* FL478-23 | 6 | 1.309 | 0.735 | - | + | - | + | ++ |
| *Microbacterium testaceum* IC37-38 | 6 | 1.190 | 0.482 | - | + | + | + | ++ |
| *Microbacterium testaceum* IC37-39 | 4 | 1.271 | 0.456 | - | + | + | + | ++ |
| *Microbacterium testaceum* IC37-40 | 4 | 1.157 | 0.515 | - | + | - | + | ++ |
| *Microbacterium testaceum* IC37-41 | 4 | 1.341 | 0.726 | + | + | - | ++ | ++ |
| *Microbacterium testaceum* IC31-45 | 6 | 1.007 | 0.417 | - | + | - | + | + |
| *Microbacterium testaceum* IR29-48 | 6 | 0.762 | 0.412 | + | + | + | + | ++ |
| *Rhizobium larrymoorei* FL478-47 | 2 | 1.003 | 0.0123 | + | + | + | + | ++ |
| *Sphingomonas pseudosanguinis* IC27-26 | 1 | 0.7053 | 0.016 | + | + | - | + | + |
| *Herbaspirillum huttiense* IC32-34 | 1 | 0.981 | 0.452 | + | + | + | - | + |

S2 Table. Continue

| **Strain** | **Salinity tolerance (%)** | **Osmotic tolerance 0.6 M sucrose** | **Osmotic tolerance 1.2 M sucrose** | **Oxidase** | **Catalase** | **Motility** | **Cellulase** | **Pectinase** |
| --- | --- | --- | --- | --- | --- | --- | --- | --- |
| *Enterobacter* sp. IC32-06 | 8 | 2.794 | 2.162 | + | + | + | + | - |
| *Flavobacterium acidificum* IC27-01 | 8 | 1.515 | 0.802 | + | + | - | ++ | ++ |
| *Flavobacterium acidificum* IC31-02 | 8 | 1.554 | 0.872 | + | + | - | ++ | ++ |
| *Flavobacterium acidificum* IC31-03 | 8 | 1.523 | 0.983 | + | + | + | + | + |
| *Flavobacterium acidificum* IC32-07 | 8 | 2.046 | 1.094 | - | + | - | + | + |
| *Flavobacterium acidificum* IR29-17 | 6 | 1.487 | 0.738 | + | + | - | + | + |
| *Flavobacterium acidificum* FL478-21 | 8 | 1.478 | 0.691 | + | + | - | + | + |
| *Flavobacterium acidificum* IC27-25 | 9 | 1.508 | 0.754 | + | + | + | + | + |
| *Flavobacterium acidificum* IC31-28 | 6 | 1.797 | 0.977 | + | + | + | + | + |
| *Flavobacterium acidificum* FL478-19 | 8 | 1.543 | 0.893 | + | + | + | + | + |
| *Flavobacterium acidificum* IC32-33 | 8 | 1.843 | 1.054 | + | + | + | + | + |
| *Flavobacterium acidificum* IC37-35 | 8 | 1.458 | 0.752 | + | + | - | - | + |
| *Flavobacterium acidificum* IR29-16 | 8 | 1.743 | 0.913 | - | + | + | + | + |

Continue

| **Strain** | **Salinity tolerance (%)** | **Osmotic tolerance 0.6 M sucrose** | **Osmotic tolerance 1.2 M sucrose** | **Oxidase** | **Catalase** | **Motility** | **Cellulase** | **Pectinase** |
| --- | --- | --- | --- | --- | --- | --- | --- | --- |
| *Kosakonia cowanii* IC32-10 | 8 | 2.361 | 1.296 | + | + | - | + | ++ |
| *Kosakonia cowanii* IC32-12 | 6 | 1.630 | 0.925 | - | + | - | + | ++ |
| *Kosakonia cowanii* IC27-24 | 6 | 2.137 | 1.149 | + | + | + | + | + |
| *Kosakonia cowanii* IC32-32 | 8 | 2.137 | 1.054 | + | + | + | + | + |
| *Kosakonia cowanii* IC31-46 | 9 | 2.227 | 1.115 | - | + | + | + | ++ |
| *Pantoea* sp. IR29-13 | 8 | 1.920 | 1.105 | + | + | + | + | + |
| *Pantoea* sp. IR29-15 | 8 | 2.226 | 1.226 | - | + | + | ++ | + |
| *Pantoea dispersa* FL478-22 | 9 | 1.525 | 0.822 | + | + | + | + | + |
| *Pantoea dispersa* IC31-29 | 8 | 1.327 | 0.436 | - | + | + | + | + |
| *Pseudomonas argentinensis* IC32-08 | 6 | 1.293 | 0.641 | + | + | + | - | - |
| *Pseudomonas parafulva* IC32-09 | 6 | 1.222 | 0.907 | + | + | + | - | + |
| *Pseudomonas oryzihabitans* IC31-04 | 6 | 1.250 | 0.759 | + | + | + | + | +++ |

Continue

| **Strain** | **Salinity tolerance (%)** | **Osmotic tolerance 0.6 M sucrose** | **Osmotic tolerance 1.2 M sucrose** | **Oxidase** | **Catalase** | **Motility** | **Cellulase** | **Pectinase** |
| --- | --- | --- | --- | --- | --- | --- | --- | --- |
| *Xanthomonas sacchari* IC31-05 | 2 | 0.609 | 0.436 | + | + | + | +++ | +++ |
| *Xanthomonas sacchari* IC32-11 | 2 | 2.358 | 0.439 | + | + | - | +++ | +++ |
| *Xanthomonas sacchari* IR29-14 | 2 | 2.261 | 1.368 | + | + | + | +++ | +++ |
| *Xanthomonas sacchari* FL478-20 | 2 | 0.831 | 0.366 | + | + | + | +++ | +++ |
| *Xanthomonas sacchari* IC31-27 | 2 | 1.681 | 0.343 | + | + | - | +++ | +++ |
| *Xanthomonas sacchari* IC32-30 | 2 | 0.552 | 0.357 | + | + | - | +++ | +++ |
| *Xanthomonas sacchari* IC31-44 | 2 | 0.682 | 0.290 | + | + | - | +++ | +++ |
| *Xanthomonas sacchari* IR29-49 | 2 | 0.598 | 0.186 | + | + | + | +++ | +++ |
| *Bacillus thuringensis* IC32-43 | 4 | 0.990 | 0.594 | + | + | + | +++ | +++ |
| *Paenibacillus hunanensis* FL478-18 | 6 | 1.633 | 1.003 | - | + | + | +++ | +++ |
| *Paenibacillus hunanensis* IC32-31 | 6 | 1.799 | 0.782 | - | + | - | +++ | +++ |
| *Paenibacillus hunanensis* IC32-42 | 4 | 1.452 | 0.607 | - | + | - | +++ | + |

(+) positive; (-) negative; For pectinase and cellulase: (+), (++), (+++), and (-) represent weak, moderate, strong and negative activity.
